# Visibility of retractions: a cross-sectional one-year study

**DOI:** 10.1186/1756-0500-6-238

**Published:** 2013-06-19

**Authors:** Evelyne Decullier, Laure Huot, Géraldine Samson, Hervé Maisonneuve

**Affiliations:** 1 Hospices Civils de Lyon, Pôle Information Médicale Evaluation Recherche, Unité de Recherche Clinique, Lyon F-69003, France; 2 RECIF, EAM Santé Individu Société 4128, Université de Lyon, Lyon F-69003, France; 3 Université Lyon 1, Lyon F-69003, France

**Keywords:** Retraction of publication, Scientific misconduct, Guidelines

## Abstract

**Background:**

Retraction in Medline medical literature experienced a tenfold increase between 1999 and 2009, however retraction remains a rare event since it represents 0.02% of publications. Retractions used to be handled following informal practices until they were formalized in 2009 by the Committee on Publication Ethics (COPE). The objective of our study was to describe the compliance to these guidelines.

**Methods:**

All retractions published in 2008 were identified using the Medline publication type “retraction of publication”. The notices of retraction and the original articles were retrieved. For each retraction, we identified the reason for retraction, the country of affiliation of the first author, the time to retraction, the impact factor of the journal and the mention of retraction on the original article.

**Results:**

Overall, 244 retractions were considered for analysis. Formal retraction notices could not be retrieved for 9. Of the 235 retractions available (96%), the reason was not detailed for 21 articles (9%). The most cited reasons were mistakes (28%), plagiarism (20%), fraud (14%) and overlap (11%). The original paper or its location was found for 233 retractions (95%). Of these, 22% were available with no mention of the retraction.

**Conclusion:**

A standard retraction form could be helpful, with a check list of major reason, leaving the editor free to provide the reader with any further information. Original articles should remain available with a clear mention of the retraction.

## Background

The process of scientific communication relies on trust: the researcher is supposed to conduct his research according to Good Practice, they should report the results properly [1] and declare any conflict of interest [2]. It is difficult for reviewers to detect errors or suspected fraud [3]. Their task is to improve the paper. Various types of inappropriate behavior can be encountered during the writing of a paper and the review process, including misconduct, faked data, falsification, ethical misconduct, plagiarism, etc. [4]. Mistakes, i.e. misinformation without intent, can also diminish the quality of scientific evidence [5]. In cases of scientific misconduct or mistakes, it is necessary that readers are informed, which is why the editor later publishes a correction (correcting a mistake by substituting correct information), expression of concern (issued in case of suspected misconduct, not yet proven) or retraction (published in case of proven misconduct) [6,7].

Following the Joachim Boldt case with 88 retractions [8] and the Scott Reuben case with 21 falsified papers [9], the subject of retraction was studied in 4 dedicated publications in 2011 [5,10-12]. The number of retractions in journals covered by the Science Citation Index Expanded has increased 20 times i.e. a tenfold increase since there has been a twofold increase in articles production between 1990 and 2008 [13]. A similar tenfold increase was found when focusing on Medline only (1999–2009), although retraction remains a rare event since it only represents 0.02% of publications [10].

The Committee on Publication Ethics (COPE) has established guidelines on dealing with retractions in 2009 [14]. COPE recommends that retractions should be issued in case of unreliable findings (misconduct or error), plagiarism or unethical research.

To better understand the reasons for retractions, 5 studies investigated cohorts of retraction notices (sometimes leading to more than one publication) [10-12,15-18]. All these studies covered different time periods, different durations of time, and provided the rate of retractions related to mistakes (research error and inability to reproduce the results). The rates of retractions related to mistakes tended to decrease according to the last year investigated: 55% for 1997 [17], 62% in 2002 [15], 42% in 2004 [16], 39% in 2008 [10] and 32% in 2010 [11].

COPE’s guidelines also state that notices of retraction should clearly identify the retracted article (title and authors) and be linked with the retracted article. The notices should be available freely and state the reason for retraction (without being defamatory) and who is retracting. Steen found that 45% of retracted papers were either available without any mention or deleted [11].

We analysed retractions published in Medline over a single year period to describe the conformity with retraction guidelines as well as the reasons for retraction and their distribution across countries.

## Methods

### Data extraction (August, 22th 2011)

All retractions published in 2008 were identified using the Medline publication type “retraction of publication”. The year 2008 was chosen to ensure that all notices of retraction to be correctly indexed at the time of the data extraction. We retrieved the retraction notice and the complete original article for all retractions. All documents were retrieved through the internet, and we did not search paper journals from the local medical library. For each retraction notice, we recorded the numbers of articles retracted, the time to retraction (number of years elapsed between publication of the original paper as noted on the pdf format, and the 2008 publication of the retraction), the country of affiliation of the first author (according to the address of the first author which is usually the same country as the last author, and often the country where the research was done), the impact factor of the journal in 2008 and the reasons for retraction. For the reason for retraction, we used the terminology agreed upon between investigators (Table 1).

**Table 1:** Reasons used to classify retractions: proposed definitions

Fraud	Falsified data, fabricated data
Inconsistent data	Confirmed doubt over data raised by others
Mistakes	Mistakes concerning data found in the paper raised by the author(s)
Plagiarism	Publication of data or text already published by others
Overlap	Multiple publication of same data or self-plagiarism
Property or legal concerns	Publication of elements without obtaining permission
Ethics	Concerns on the ethical validation of the research
Authorship	Disputed authorship
Editor	Production or administrative error

Data were extracted by GS, then LH and ED independently reviewed the reasons for retractions. Discrepancies were resolved by consensus between the two reviewers.

### Analysis

We described the frequency of each reason, and cross-tabulated it with the country to obtain the ranking of countries for each reason.

Among the 9 reasons, we combined fraud, mistakes and inconsistent data since retracted articles led to the circulation of false information (labelled as “false information”) and were compared to the 6 other reasons combined.

To assess the conformity of visibility of retraction to good practices, we decided to choose the COPE guidelines. Even if published in 2009, COPE guidelines were the formalization of common sense and informal practices of some journals. We therefore determined whether the retraction was mentioned on the pdf of the original article or not. Visibility of retractions was defined by three possibilities: 1) deletion of the article from the journal’s website; 2) presence of the article with a mention of the retraction on the paper itself (either a comment at the beginning and/or at the end; or a clear indication for example printed diagonally across the paper) and/or mention on the journal’s website; and 3) presence of the article with no mention.

Time to retractions and impact factors were compared using the Mann–Whitney or Kruskal-Wallis test. Visibility of retractions was compared using Chi^2^ test (or Fisher exact test where Chi^2^ test conditions were not fulfilled).

All analyses were performed using SAS v9.2 (Cary, North Carolina).

## Results

Overall, 241 notices of retraction were retrieved. Concerning access, 209 were available by open access or accessible through our institution’s subscription, 23 were found outside of our institution and 9 were impossible to locate.

These 241 notices of retraction represented 253 retractions (10 retracting 2 articles and 1 retracting 3 articles). After deleting 3 duplicate notices, 250 retractions remained available. Lastly, one “retracted retraction” and 5 partial retractions were discarded. Overall, 244 retractions were considered for analysis.

### Reason for retraction (n = 235)

As previously stated, formal retraction notices could not be retrieved for 9 of the retractions. Of the 235 retractions available, the reason was not given for 21 articles (9%). For example, the only information given by some journals is that the article is being retracted. Sometimes, it is also specified “retracted by the authors” or “retracted to be consistent with the publisher policy on article withdrawal” or “retracted by the authors with the agreement of the journal editors and the publisher” without any other details.

The most cited reasons were mistakes (n = 65, 28%), plagiarism (n = 48, 20%), fraud (n = 34, 14%) and overlap (n = 25, 11%) (Table 2).

**Table 2:** Reasons for retraction ranked according to countries with at least 10 retractions in 2008

	**Total**	**Mistakes**	**Plagiarism**	**Fraud**	**Overlap**	**Not detailed**	**Authorship**	**Inconsistent data**	**Property or legal concerns**	**Editor**	**Ethics**
**Total**	235	65	48	34	25	21	12	11	8	8	3
USA	52	**31 (48)**	2 (4)	3 (9)	5 (20)	**4 (19)**	1 (8)	**3 (27)**	**2 (25)**	1 (13)	.
India	35	2 (3)	6 (13)	**15 (44)**	**8 (32)**	1 (5)	1 (8)	1 (9)	.	1 (13)	.
China	29	7 (11)	**10 (21)**	.	2 (8)	3 (14)	**3 (25)**	1 (9)	**2 (25)**	1 (13)	.
Japan	25	3 (5)	2 (4)	12 (35)	2 (8)	2 (10)	.	2 (18)	.	.	**2 (67)**
United Kingdom	11	3 (5)	4 (8)	1 (3)	.	2 (10)	.	1 (9)	.	.	.
Korea	11	5 (8)	.	1 (3)	.	1 (5)	.	.	**2 (25)**	**2 (25)**	.

Values are n (column percentage). Column percentage = number of given reason in a country/ total for this reason; allowing to see which countries contribute the most to each reason.

The column percentages do not add to 100% because some countries were not reported in the table.

The reasons are also presented according to countries in Table 2. In India and Japan, the most frequent reason was fraud, representing 43% for India (15/35) and 48% for Japan (12/25). In China, it was plagiarism with 34% (10/29); whereas in the USA the main reason was mistakes at 60% (31/52) and plagiarism at 36% for United Kingdom (4/11).

Time to retraction and impact factors are compared between fraud, mistakes and plagiarism in Table 3.

**Table 3:** Comparison of the 3 main reasons for retraction by impact factor, time to retraction and visibility of retractions

	**Fraud**	**Mistakes**	**Plagiarism**	**p-value**
**Impact factor (n = 137)**	4.9	7.2	2.3	<0.0001
	(3.2, 2.9-14.6)	(4.1, 0.7-31.4)	(1.9, 0.3-9.8)	
**Time to retraction (n = 244), years**	2.2	2.5	3.2	0.722
	(2, 0–8)	(2, 0–10)	(2, 0–10)	
**Publication year of retraction *vs *original article (n = 244), n (%)**				0.024
same year	2 (6)	11 (17)	8 (17)	
1 to 5 years	31 (91)	47 (72)	29 (60)	
>5 years	1 (3)	7 (11)	11 (23)	
**Visibility (n = 233), n (%)**				0.057
mention	28 (82)	38 (62)	22 (51)	
no mention	5 (15)	15 (25)	12 (28)	
deletion	1 (3)	8 (13)	9 (21)	

Values are mean (median, min-max) unless otherwise specified.

### Mention of retraction on the original article (n = 233)

Of the 244 original articles, 11 could not be retrieved because they had to be paid for online or were printed articles or impossible to locate. We therefore found the original paper or its location for 233 retractions (95%). Of these 233 retracted original articles, 139 (60%) were available with a mention of the retraction either on the article or on the journal’s website, 52 (22%) were available with no mention and 42 (18%) had been completely deleted (the publication concerned was no longer available on the journal’s website) (Table 4). For articles with at least one mention, 101 (73%) had a mention both on the article and the website, for 22 (16%) the mention only featured on the website, 14 (10%) only on the article and the 2 last cases (1%) were 1) an empty pdf marked “retraction” with no mention on the website and 2) an article with a mention on the website for which the paper was no longer available in pdf format but which was available as html text. For the 115 papers which mentioned the retraction on the original article, 80 (70%) had a clear indication and 35 (30%) had notes at the beginning and/or at the end of the paper (Table 4).

**Table 4:** Mention of retraction on the original article

	**n (%)**
**Original article (n = 233):**	
Deletion	42 (18)
No Mention	52 (22)
Mention	139 (60)
*both website and article*	*101 (43)*
* article*	*14 (6)*
*website*	*22 (10)*
other*	*2 (1)*
**If mention available on article (n = 115), details:**	
At least a clear indication†	80 (70)
*present without notes*	*72 (63)*
*present with notes at the end*	*5 (4)*
*present with notes at the beginning*	*3 (3)*
Notes only	35 (30)
*at the end*	*9 (8)*
*at the beginning*	*19 (16)*
*both at the beginning and the end*	*7 (6)*

* One empty pdf marked “retraction” with no mention on the website and one article with a mention on the website for which the paper was no longer available in pdf format but which was available as html text.

† Clear indication is for example printed diagonally across the paper.

Visibility of retraction on original articles was significantly higher for false information (p = 0.003). Among the 106 articles with false information, 75 (71%) had a mention compared to 64 / 127 articles (50%) retracted for other reasons; the rate of deleted articles was lower for false information compared to other reasons (11 (10%) vs 31 (24%)); finally, the rate of articles available with no mention was similar (20 (19%) vs 32 (25%)).

## Discussion

The most frequent reasons for retraction in 2008 were mistakes (28%), followed by plagiarism (20%) and fraud (14%), and journals insufficiently followed the retraction good practices formalized by the 2009 COPE guidelines.

All investigations into Medline retractions have highlighted the fact that it is difficult to find formal retraction notices containing the reason for retraction (studies found that formal explanation was unavailable for 5% [10], 11% [15], 12% [16], 18% [11]). Here, the content of the retraction was not explicit for 21 retractions (9%). As recommended in previous studies, editors should always provide the precise reason for retraction. Sometimes obtaining the formal retraction notice is subject to a pay-per-view fee, and is therefore linked to the institution’s membership. One study specified that the search was restricted to articles accessible within the institution [10], another specified that 46 notices (5.8%) could not be retrieved [11], the other studies did not specify these cases were excluded or counted them as “reason unclear”. Overall, in our study, 9 retractions were not available even with payment. The fact that retraction notices are not freely available and when available, are not explicit, contravenes the COPE guidelines [14] and should be a priority for journals as they are essential for the reader’s understanding of whether the results still hold.

However, editors are not always willing to retract articles [19]. It is therefore necessary that all journals implement a retraction policy, indeed in a 2004 review it was shown that only 18% had a dedicated policy [20]. The way to handle mistakes is sometimes controversial: some recommend to publish corrections [7], whereas others state that they should be full retractions [14]. Practices are not homogeneous [21], and retractions for mistakes are regularly published.

It has already been demonstrated that the retracted literature continues to be cited [18,22]. One of the explanations might be that retractions are not correctly reported on original article. We found that the mention of the retraction was incorrect for half of the retractions: the article was either completely deleted, or was still available with no mention. This is similar to the 45% found by Steen [11]. The retraction good practices formalized by the COPE guidelines [14] are probably not well known or respected. Clear rules should be applied to ensure that this literature is identified as retracted. To be consistent with full transparency of publication, it is inappropriate to completely remove a retracted article from the website of a journal. As long as a clear mention of the retraction is available on both the journal website and the article, scientists must still have access. Some publishers are probably afraid of the circulation of false information, particularly of fraudulent data, and retracted articles continue to be removed from printed or online versions of some journals.

We also decided for the purposes of this study to combine fraud, inconsistent data and mistakes to estimate what proportion of retracted articles might have damaged the body of knowledge. Other reasons include, of course, misconduct however the information in these papers remains true. Steen has also shown that many patients are put at risk since approximately 2 472.6 subjects per retracted paper were enrolled in subsequent studies using this retracted research [23]. Recently, the CrossMark system has been developed following the collaboration between several publishers to standardize the way of providing readers how to locate the current version of an article [24]. If one journal applied the CrossMark icon on every pdf, it is to try to maintain the content of published articles and, by clicking on it, to warn readers if changes have occurred. In the future, this system may reduce citations to retracted articles.

We cross-tabulated reasons for retraction with countries. However, retraction rates by country should be interpreted relative to publication rate: the USA represents 22% of retractions but also represents the highest number of publication. The aim of this study was to provide a global description of retraction, and due to the low numbers in each category we did not provide any statistical comparison of countries. Good practices should be better disseminated to authors and editors in order to educate them and raise their awareness to better prevent misconduct; however fraud will not be deterred by this dissemination. We found similar rates as Wager [10]: 28% for mistakes vs. 28%, 20% for plagiarism vs. 16% and 14% for fraud vs. 11%.

We noticed an association between impact factor and reasons for retraction with mistakes being published in higher impact factor journals. This could be explained by the fact that some authors might hurry to publish in a prestigious journal without taking enough time to check their data. When a paper deals with a hot topic, journals tend to publish special issues very rapidly. Moreover, mistakes are also very frequent in the USA where researchers can be subject to greater pressure and are more inclined to submit to prestigious international journals. The publications by Steen and Fang also found that higher impact factor journals were more prone to retraction [12,25].

## Conclusion

It would be useful to use a standard retraction form with for example a check list of major reason, which would then leave the editor free to provide the reader with any further information. Original articles should remain available with a clear mention of the retraction, and not only a mention on the journal website or in notes at the beginning or end of the article. This is probably on the COPE agenda, and our work can contribute to proposing definitions.

## Competing interest

The authors declare that they have no competing interests.

## Authors' contributions

ED declares that she designed the study, assessed the retractions, analysed the data, interpreted the data and wrote the manuscript. LH declares that she designed the study, assessed the retractions, interpreted the data, participated in drafting the manuscript and that she has read and approved the final version. GS declares that she retrieved the retraction notices, assessed the retractions, participated in drafting the manuscript and that she has read and approved the final version. HM declares that he participated in analysis planning, interpreted the data, participated in drafting the manuscript and that he has read and approved the final version. All authors read and approved the final manuscript.

## Authors’ information

ED is a senior researcher (PhD), LH is a senior researcher (PharmD, PhD), GS is a junior researcher (PharmD), HM is a senior researcher (MD).
